# CD133-Functionalized Gold Nanoparticles as a Carrier Platform for Telaglenastat (CB-839) against Tumor Stem Cells

**DOI:** 10.3390/ijms23105479

**Published:** 2022-05-13

**Authors:** Elham Poonaki, Ann-Christin Nickel, Mehdi Shafiee Ardestani, Lars Rademacher, Marilyn Kaul, Evgeny Apartsin, Sven G. Meuth, Ali Gorji, Christoph Janiak, Ulf Dietrich Kahlert

**Affiliations:** 1 Department of Neurology, Faculty of Medicine, Heinrich-Heine-University, 40225 Düsseldorf, Germany; elham.poonaki@uni-duesseldorf.de (E.P.); svenguenther.meuth@med.uni-duesseldorf.de (S.G.M.); 2 Institut für Anorganische Chemie und Strukturchemie, Heinrich-Heine-University, 40204 Düsseldorf, Germany; lars.rademacher@hhu.de (L.R.); makau113@uni-duesseldorf.de (M.K.); 3 Molecular and Experimental Surgery, University Clinic for General-, Visceral-, Vascular- and Transplantation Surgery, Faculty of Medicine, Otto-von-Guericke-University, 39120 Magdeburg, Germany; 4 Clinic for Neurosurgery, Heinrich-Heine-University, 40225 Düsseldorf, Germany; ann-christin.nickel@hhu.de; 5 Department of Radiopharmacy, Faculty of Pharmacy, Tehran University of Medical Sciences, Tehran 1416634793, Iran; shafieeardestani@sina.tums.ac.ir; 6 Institute of Chemical Biology and Fundamental Medicine SB RAS, 630090 Novosibirsk, Russia; eapartsin@gmail.com; 7 Laboratoire de Chimie de Coordination CNRS, 31400 Toulouse, France; 8 Epilepsy Research Center, Department of Neurosurgery and Department of Neurology, Westfälische Wilhelms-Universität, 48149 Münster, Germany; gorjial@uni-muenster.de; 9 Shefa Neuroscience Research Center, Khatam Alanbia Hospital, Tehran 9815733169, Iran

**Keywords:** cancer stem cells, nanomedicine, glutaminase, onco-metabolism, CD133

## Abstract

The failure of a long-lasting curative therapeutic benefit of currently applied chemotherapies against malignant cancers is suggested to be caused by the ineffectiveness of such interventions on cancer stem cells (CSCs). CD133/AC133 is a cell surface protein previously shown to have potential to identify CSCs in various tumors, including brain tumors. Moreover, an increase in the rate of cellular metabolism of glutamine and glucose are contributors to the fast cellular proliferation of some high-grade malignancies. Inhibition of glutaminolysis by utilizing pharmacological inhibitors of the enzyme glutaminase 1 (GLS1) can be an effective anti-CSC strategy. In this study, the clinical-stage GLS1 inhibitor Telaglenastat (CB-839) was loaded into PEGylated gold nanoparticles equipped with the covalently conjugated CD133 aptamer (Au-PEG-CD133-CB-839) and exposed to a collection of CD133-positive brain tumor models in vitro. Our results show that Au-PEG-CD133-CB-839 significantly decreased the viability of CD133-postive cancer cells in a dose-dependent manner, which was higher as compared to the effects of treatment of the cells with the individual components of the assembled nanodrug. Interestingly, the treatment effect was observed in glioblastoma stem cells modeling different transcriptomic subtypes of the disease. The presented platform is the fundament for subsequent target specificity characterization and in vivo application.

## 1. Introduction

Glioblastoma (GBM) is a malignant brain tumor in adults, with low survival time and excessive potential for recurrence [1]. Regarding different studies, the insufficient effectivity of common treatments in GBM might be correlated with the existence and biochemical capacities of GBM stem cells (GSCs) [2,3]. Moreover, the potency of GSCs can be associated with their differentiation into endothelial cells, leading to stimulating angiogenesis in glioblastoma [4]. In this regard, CD133-positive GSCs predominantly promote the angiogenesis and ability of proliferation compared to CD133-negative GSCs [5]. CD133 is a glycoprotein antigen and dominant biomarker for the recognition of glioblastoma stem cells due to overexpression and plays a crucial role in the oncogenic potential of GSCs [6,7]. Therefore, targeting GSCs via CD133 can be effectively applied for eradicating GSCs and their tumorigenic capacities [8,9]. On the other hand, recent reports have demonstrated the crucial role of glutamine as an abundant nitrogen and carbon source instead of glucose in cancer cell growth including GSCs [10,11].

The glutamine synthesizes ATP and nucleotides in the plasma. Glutaminase 1 (GLS1), as a mitochondrial enzyme, is required for metabolizing glutamate and ammonia from glutamine and its activity has been shown to be upregulated in tumor cells compared to normal cells. The effectiveness of pharmacological GLS inhibition as a potential strategy to eradicate GSCs is well-established in many studies including our previous work [12,13]. We previously identified CB-839 to act specifically on the putative target of the enzymatic activity of GLS1 and utilize a higher anti-growth effect on tumor stem cells as compared to non-cancer cells such as neural stem cells.

Establishing efficient brain cancer therapies still remain complicated and challenging due to the function of the blood-brain barrier (BBB) as an obstacle in passing systemically administered therapeutic agents to the central nervous system [14]. Therefore, selecting appropriate carriers considering the size and other determinant parameters is a critical approach to achieving an optimal therapy to overcome the BBB for the successful transport of therapeutic agents [15]. In comparison with conventional chemotherapeutic drugs, applying nanoparticles for delivery of such substances is widely used due to some outstanding advantages, including small size, lower immunogenicity, enhanced permeability and retention effects [16,17,18]. Furthermore, nano-based drug delivery systems are also effective in extending the half-life of medicines in clinical applications regarding higher stability as well as decreasing the resistance of cancer therapeutic agents [18,19,20].

Accordingly, gold nanoparticles (AuNPs) are one of the extensively studied inorganic NPs and are considered a promising nanomaterial for cancer treatment, especially in GBM regarding its biocompatibility and small size for the high permeability of the BBB and biocompatibility. Modified AuNPs are able to provide a widely accepted strategy for precise drug delivery approaches [21,22]. However, due to their susceptibility to agglomerate, modification of the surface with protecting polymers such as poly ethylene glycol (PEG) can effectively increase the stability as well as their functions and biocompatibility [23]. PEG is a water-soluble biocompatible polymer, which can prevent gold nanoparticles from aggregation and recognition by the immune system [24].

**Figure 1:**
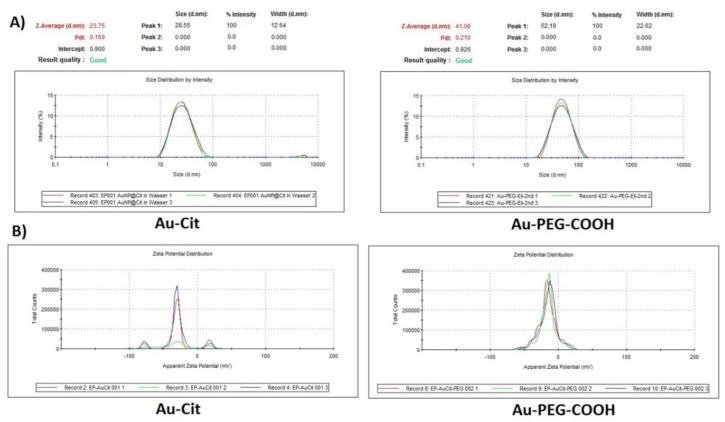
Characterization of gold nanoparticles. (**A**) DLS and (**B**) zeta potential of Au-Cit and Au-PEG-COOH in aqueous solution. The comparison shows that no aggregation occurs upon PEGylation of the citrate-covered AuNPs.

In the present study, a 15-nucleotide base-pair CD133 aptamer targeting glycoprotein antigen CD133/AC133 in GSCs was conjugated to our Au-PEG-CB-839 platform in order to improve the targeting of cancer stem cells (Figure 1). The surface modification of gold nanoparticles by PEG increased the stability of our nanoparticles and assisted in the conjugation of the CD133 aptamer and the loading of CB-839, compared to the citrate-covered AuNPs. In this study, AuNPs were synthesized by reduction of KAuCl4 with sodium citrate and first modified with carboxy-thiol-terminated PEG. Subsequently, the Au-PEG-COOH particles were conjugated with commercially available RNA aptamer to Au-PEG-CD133. Finally, CB-839 was loaded to yield Au-PEG-CD133-CB-839, which was evaluated concerning the survival of human GSCs (GBM1, NCH644) and classical human glioma cells (U87 and LN229).

## 2. Results

### 2.1. Characterization of AuNPs and Au-PEG-COOH

The synthesis of AuNPs as a nanocarrier was done by using KAuCl_4_ with a particular ratio of sodium citrate due to its specification as a reducing agent based on the Turkevich method [25]. The characterization of AuNPs and Au-PEG-COOH was assessed by dynamic light scattering (DLS) and transmission electron microscopy (TEM) analysis. From the DLS and Zeta potential curve for both Au citrate coated and PEGylated gold-coated citrate and Au-PEG-COOH at pH = 4.6, a hydrodynamic diameter of 29 ± 12 and 52 ± 23 nm, respectively, was determined. The small increase in size is due to the coverage with the polymer but no detectable aggregation takes place upon PEGylation which otherwise would have resulted in much larger sizes (Figure 1).

After PEGylation of the gold nanoparticles with carboxy-PEG-thiol, the stability of Au-PEG-COOH was investigated and compared to Au-Cit in different solutions relevant to the current reactions. This could assess the efficacy of utilizing PEGylating gold nanoparticles before conjugation with the CD133 aptamer as well as following the loading of CB-839. In these experiments, different solutions such as phosphate-buffered saline (PBS) with pH and concentrations regarding the reactions were assessed (Figure S1A). In addition, CB-839 could only be dissolved in dimethyl sulfoxide (DMSO) and is insoluble in water and ethanol. Thus, the stability of gold nanoparticles and Au-PEG-COOH in DMSO was evaluated (Figure S1C). The results demonstrated, especially in PBS, that PEG prevents agglomeration of the AuNPs. Consequently, Au-PEG was used as the platform for conjugating to the aptamer and loading the drug (Figure S1). These data could approve the logical reason for applying a polymer such as PEG for making gold nanoparticles more stable instead of utilizing naked gold nanoparticles for conjugating and loading any medicines.

### 2.2. The Evaluation of Conjugation CD133 Aptamer to Au-PEG-COOH

**Figure 2:**
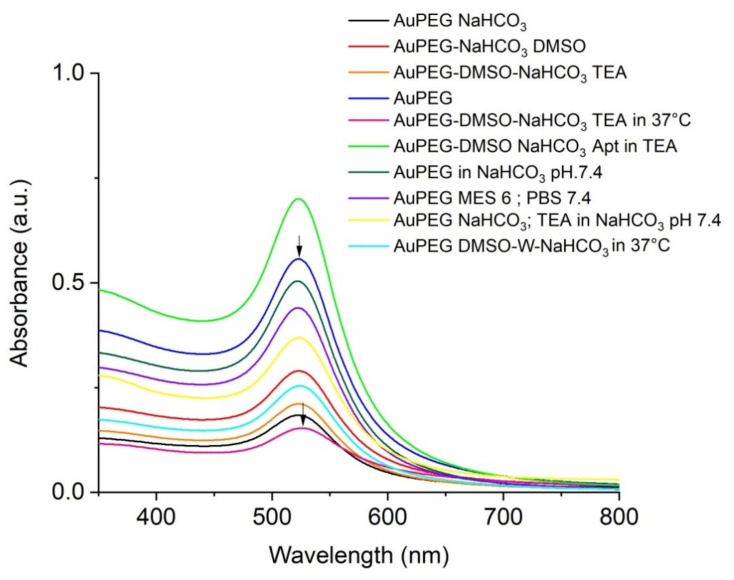
The results of different protocols were used for conjugating CD133 aptamer to Au-PEG-COOH. These graphs have demonstrated different wavelengths of all reactions and one shifting to the right in one of the reactions compared to Au-PEG as our control. This shifting has shown the covalent reaction which happened to the nanoparticle after conjugating with the CD133 aptamer.

The Au-PEG-COOH can be covalently bound to the amine (NH_2_) group of the 15mer CD133 aptamer (sequence: 5′-NH_2_-CCCUCCUACAUAGGG-3′) through amide (-CO-NH-) formation. The absorption wavelength of surface plasmon resonance of the PEGylated AuNPs was approximately 520 nm (Figure 2). The covalent reaction between NH_2_ of the aptamer with COOH of Carboxy-PEG-thiol at the gold nanoparticles caused a small shift of 5 nm to longer wavelengths of the UV-VIS absorption. Different synthetic protocols were followed to ensure aptamer conjugation. For example, Au-PEG-COOH was reacted with CD133 in a 50:50 DMSO water solution where the pH was set with NaHCO_3_ to pH 7.4. DMSO and triethylamine (TEA) were used in order to prevent hydrolysis of 1-ethyl-3-(3-dimethylaminopropyl) carbodiimide (EDC) and N-hydroxysulfosuccinimide (sulfoNHS). After activation of the carboxyl group of Au-PEG-COOH, aptamers diluted in NaHCO_3_ pH: 8.5 were added to the reaction for 2 h in the shaker incubator at 37 °C, 350 rpm for 30 min. After centrifugation of Au-PEG-Apt and suspension in DMSO, the result has shown appropriate shifting in UV-VIS absorbance based on the covalent reaction between COOH of PEG and NH_2_ of the aptamer (Figure 2). Consequently, the results have shown a slight shift of approximately 5 nm to the right side and exhibited the desirable conjugation.

In another eight synthesis protocols we evaluated the influence of pH and temperature to achieve the appropriate result for aptamer conjugation. We examined MES buffer with pH: 6 and NaHCO_3_ pH: 7.4 to utilize EDC and sulfoNHS regarding their optimal pH in relevant texts for their activity, respectively, at room temperature (RT). In another experiment, the use of MES buffer pH: 6 and PBS: 7.4 in the time of EDC and sulfoNHS activity was examined at RT. Moreover, in different conditions and according to the sensitivity of aptamers to heating, we just performed the reaction in a 37 °C shaker incubator, which was effective. Since EDC and sulfoNHS are prone to hydrolysis, our results have shown that decreasing water in the reaction could be beneficial to amend the carbodiimide chemistry. Therefore, in our final protocol, it was attempted to minimize water in the reaction as much as possible. This is important to note that due to the sensitivity of the aptamer as an RNA sequence, all reactions were done in RNAase DNAase conditions, although the manufacturer’s modifications almost protected our aptamer from denaturing the sequences (Figure 2).

**Figure 3:**
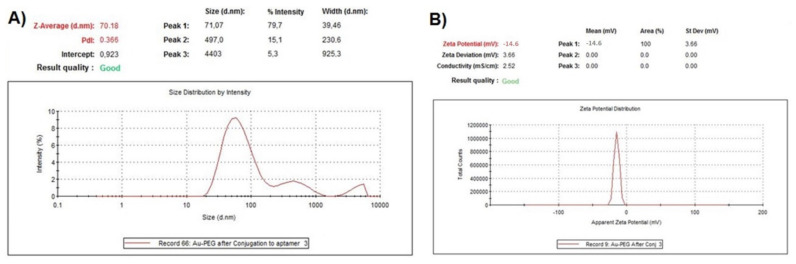
Characterization of Au-PEG-CD133 (**A**) DLS with a significant change in the size and without any agglomeration and (**B**) Zeta potential with no significant changes, which was expected.

Characterization of conjugated gold nanoparticles also has been examined by DLS with significant changes in size compared to Au-PEG-COOH without agglomeration and Zeta Potential with no dramatic changes having been observed as our expectation (Figure 1 and Figure 3). The Zeta potential is determined by the negative charge of the partially deprotonated carboxyl (-COOH) groups of citric acid, Carboxy-PEG-thiol and the phosphate groups of the oligonucleotides of CD133 within their acid-base equilibria of Au-Cit, Au-PEG-COOH and Au-PEG-CD133, respectively.

### 2.3. TEM Analysis

**Figure 4:**
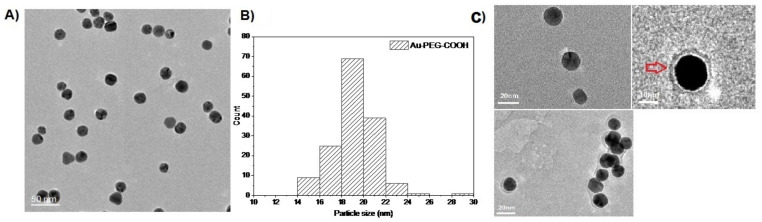
TEM images of (**A**) Au-PEG-COOH nanoparticles. (**B**) Histogram for the size distribution of Au-PEG-COOH. (**C**) Au-PEG-CD133 particles. There is an organic halo around the gold core which may be due to PEG or PEG-CD133.

Transmission electron microscopy showed the diameter of the gold core of the Au-NPs between 8 and 15 nm compared to PEGylated gold nanoparticles with about 19 ± 2 nm (Figure 4). TEM also demonstrated an organic halo around the gold core of the nanoparticles, yet at the given resolution, TEM does not allow to differentiate the organic halo further into PEG or PEG-CD133 (Figure 4C).

### 2.4. Analysis of CB-839 Loading to Au-PEG-CD133

**Figure 5:**
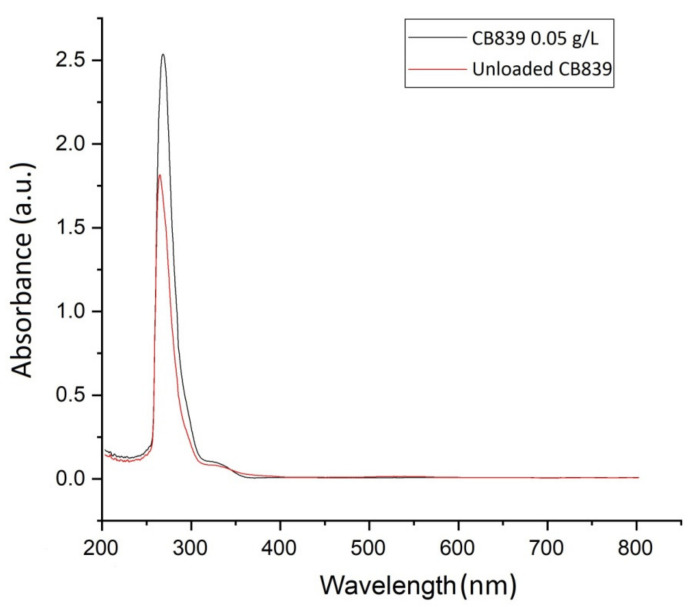
Comparison of absorbance standard concentration of CB-839 (0.05 g/L) with unloaded CB-839 in the supernatant. The percent of drug loading efficiency was calculated by the formula based on λ_max_ of absorbance, which was approximately 30%.

After conjugation of CD133, the amount of CB-839 loaded to Au-PEG-CD133 was evaluated based on the calculation of loaded and unloaded nanoparticles. In this regard, the supernatants were collected after centrifugation and utilized to quantify the unloaded amount of CB-839, which remained in solution via UV-VIS compared to the amount of CB-839 in the stock of 0.05 g/L. The percentage of drug loading efficiency (DLE) was calculated via the formula below and based on λ_max_ and the area of the graph in UV-VIS spectra (Figure 5). Regarding the amount of absorbance of the standard stock solution of CB-839, which was loaded to nanoparticles compared to the amount of concentration of CB-839 in the stock. In this regard, the percentage of DLE was approximately 30%.

### 2.5. Functional Evaluation of Drug Carrier Platform on Tumor Models In Vitro

**Figure 6:**
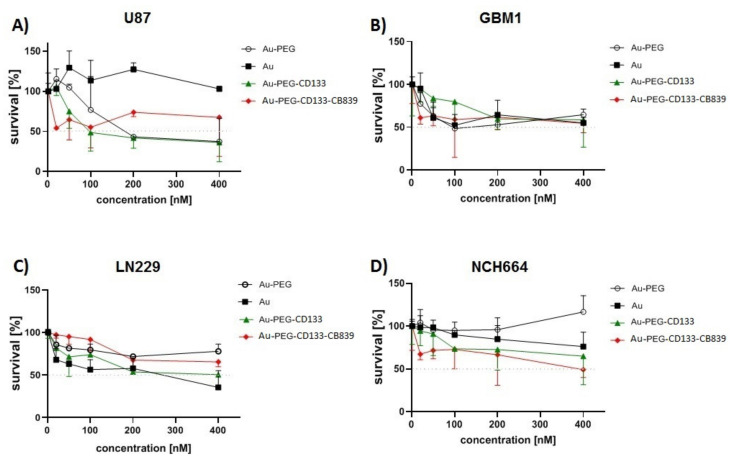
Cytotoxic effects of Nano-CB-839 on GSCs and GBM cells. These results exhibited the inhibitory efficacy of Au-PEG-CD133-CB-839 as well as other similar compounds in different concentrations after 24 h. Regarding the obtained data, the concentration of 20 nM was selected as a lower concentration close to IC50 to treat the GCSs and GBM cells for the main analysis of cell proliferation.

Following the development of nanomedicine, anti-growth effects of Nano-CB-839, as well as corresponding control conditions (such as naïve components of carrier platform and payload), have been assessed by retrieving IC50 values on GBM cells and GSCs. IC50 of nanomedicine was determined in different concentrations. The concentration of 20 nM, which was the lowest level of nanomedicine close to IC50 with the highest mortality in all GSCs and GBM cells, has been chosen for other treatments (Figure 6).

### 2.6. FACS Based Quantification of Target Cell-Surface Expression on Cell Models

**Figure 7:**
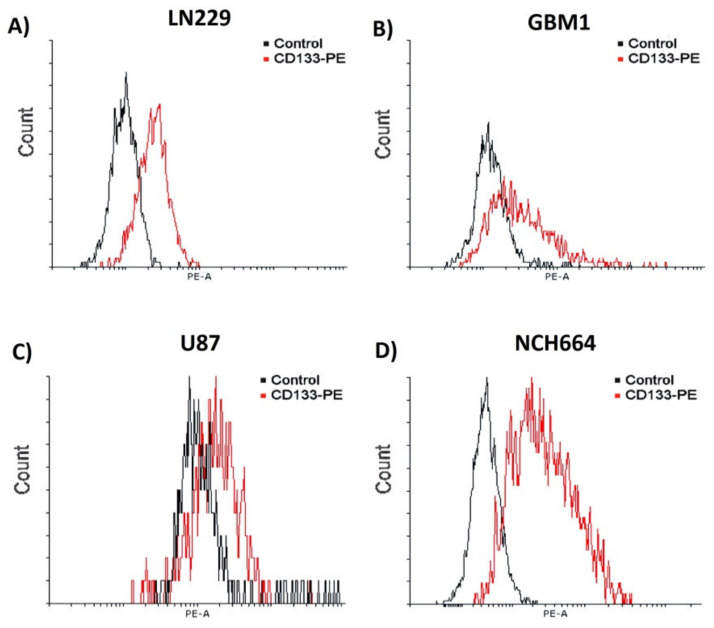
Analysis of the amount of CD133 expression on glioblastoma cancer cells by FACS. Depicted are histograms of exemplary measurements of cells stained with CD133/AC133-PE. The cells have displayed different ratios of CD133 expression (**A**) LN229 = 33.8%, (**B**) GBM1= 33.1% (**C**) U87 = 21.5% and (**D**) NCH664 = 89.2%.

All the studied cell models, including classical cell systems U87, LN229, and NCH664, exhibited CD133+ cell populations to different extents. The highest and lowest quantity of expression in CD133+ cells referred to NCH664 and U87 with 89.2% and 21.2% respectively (Table 1, Figure 7).

### 2.7. Effect of Nanodrug Exposure on Cellular Viability

**Figure 8:**
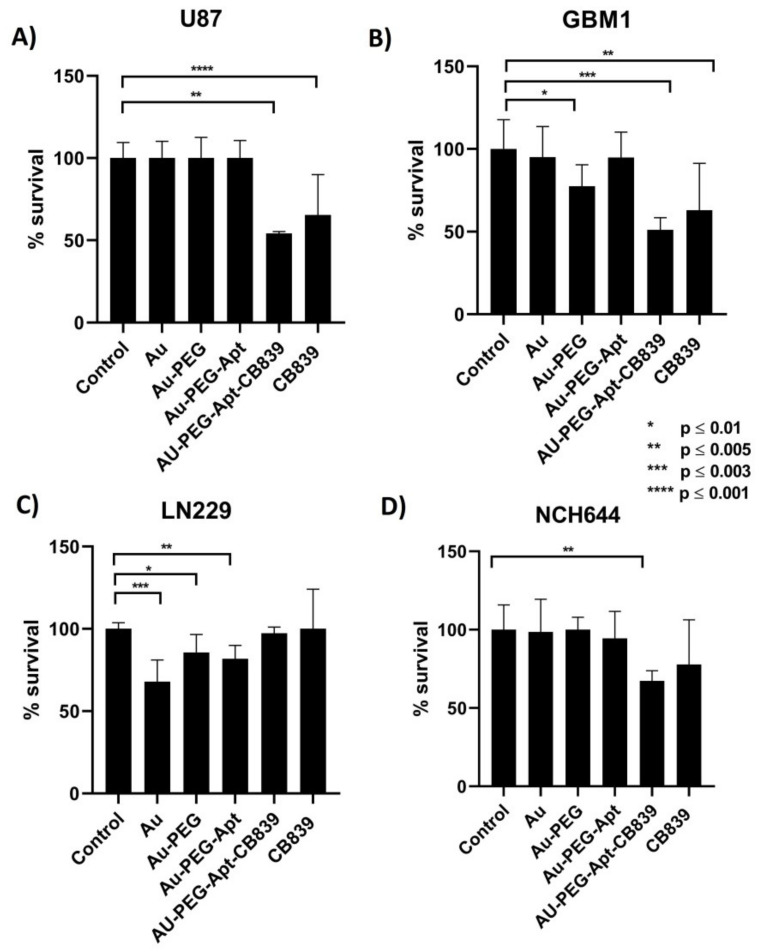
MTT assay in GSCs and GBM cells; (**A**) U87, (**B**) GBM1, (**C**) LN229, and (**D**) NCH644. The majority of groups have been affected considerably by Au-PEG-CD133 and Au-PEG-CD133-CB-839 compared to Au and Au-PEG. The results have shown the significant efficacy of Au-PEG-CD133-CB-839 on most cohorts (GBM1, U87and NCH-644) in concentrations of approximately 20 nM, which could show the appropriate efficiency of our nanomedicines in low concentration compared to naïve CB-839, Au-Cit and Au-PEG-COOH as our control groups of nanomedicines. The cell line LN229 exhibited high resistance to GLS1 inhibition interventions.

Thiazolyl Blue Tetrazolium Bromide (MTT) assay was performed to evaluate the functional effects of nanomedicine on the viability glioblastoma cells in the exposure of the developed nanodrug (Au-PEG-CD133-CB-839) and controls (Au-Cit, Au-PEG-COOH, native CB-839, Au-PEG-CD133) in the previously established concentration of 20 nM (Figure 8). Strikingly, in most of the cell lines, a significant strong anti-survival effect of Au-PEG-COOH-functionalized CD133 nanoparticle loaded by CB-839 could be observed compared to other compounds. Moreover, Au-PEG-CD133-CB-839 could suppress nearly 50% of cell growth in the concentration of 20 nM. In conclusion, Au-PEG-CD133-CB-839 was considerably efficient in most glioblastoma cells, although LN229 showed resistance to the medicine in this treatment.

## 3. Discussion

Inorganic nanocarriers such as gold nanoparticles are promising systems for drug delivery to tumors based on their stability, lower toxicity higher loading capacity, and biocompatibility [26]. Glutamine is a major nitrogen source with a crucial role in cancer cell growth, including in the progression of glioblastoma [27,28]. GLS1 presents a druggable therapeutic target via GLS1 inhibitors, in particular CB-839 contributed to the depletion of GSCs and glioblastoma cell growth according to the lack of nutritional supplements resulting from the reduction of glutamine penetration into the TCA cycle. Antitumor activity of CB-839 in different types of cancer, including GBM has been shown to significantly decrease in proliferation, self-renewal, and migration of cancer cells [12,29]. Nevertheless, clinical investigations exhibited considerably lower brain accumulation of CB-839 compared to other therapeutic agents due to the limited permeability of the BBB [30]. Furthermore, the indicated toxicity of native GLS inhibitors restricted their therapeutic application in different clinical studies [31]. Thus, employing appropriate low toxic carriers to reduce the adverse effects of clinical GLS inhibitors and to improve their bioavailability at the intended target site are of critical need. Additionally, combination therapy or utilization with other components such as PEG has been recommended aiming to gain appropriate therapeutic output and prevention of unacceptable toxicity [32,33,34,35]. Our project contributes to these efforts by developing an inorganic carrier platform with a molecular-targeting moiety.

PEG has been applied for surface modification of inorganic agents in an effort to achieve effective drug delivery to cancer cells [36]. Although utilizing other materials such as polyvinyl alcohol (PVA), polaxamer, and polysaccharides have been noticed, PEG has still prevailed as a commonly used material due to its considerable advantages. It has been demonstrated that the high affinity of gold nanoparticles to thiol groups leads them to proper characteristics as well as enhancing penetration of the BBB and escaping from macrophage uptake in brain delivery followed by modification with carboxy-PEG-thiol [37,38]. PEGylation of gold nano-platforms not only avoids undesired protein interactions in biological environments but also concludes with EPR effects and more intracellular accumulation [39]. Surface functionalization of nanoparticles is also a potent technique to overcome hurdles in cancer therapy resistances, such as target tissue and target cell bioavailability [40,41,42]. Utilizing RNA or DNA aptamers is one of these strategies in order to develop functionalized nanoparticles. The principal cause of exploiting aptamers instead of conventional antibodies is based on their stronger binding tendency to the small molecules (such as gold), as well as higher stability, appropriate shelf life, and no immunogenicity. In 2004 the first RNA aptamer was approved for clinical use by the US FDA and since then 11 additional aptamers have entered clinical testing for different therapeutic implications, however, until now no aptamer–assisted cancer therapy is market-available [43]. Indeed, the usage of RNA aptamers is in such progress that even one of them has been approved by FDA recently and was reported in several areas of literature [44,45,46]. In this project, we have followed the mentioned research and incorporated validated CD133-targeting aptamers (45-46) as our AuNP-targeting moiety.

Our data are a continuation of our cancer stem cell target discovery and target validation research identifying GLS1 as a very prominent and druggable target. We previously showed that CB-839 is particularly worth further investigation compared to other GLS1 inhibitors, as it shows most anti-cancer cell potency meanwhile reducing off-target effects on non-cancer stem cells [12]. Moreover, we improved our established synthesized AuNP carrier platform for GLS1 delivery. In our previous study, we could not load more than 4% of CB-839 on PEGylated gold nanoparticles despite further advantages of PEG for drug delivery compared to PVA, which was mentioned above. Therefore, in our current study, we attempted to ameliorate our methods such as employing a higher molecular weight of PEG along with some modifications in the chemical reaction process, leading to successfully loading of CB-839 at approximately 30% around Au-PEG-CD133 particles. Regarding several investigations, choosing the appropriate molecular weight of PEG is a crucial issue to provide an adequately rich layer surrounding nanoparticles [24].

Additionally, aggregation of gold nanoparticles could occur with a low molecular weight of PEG. The optimal molecular weight of PEG of at least 5000 Da has been emphasized in previous investigations in order to attain higher stability, prevention of agglomeration, and prolong blood circulation of nanomedicines [47,48]. Hence, the carboxy PEG thiol terminated with MW (5 kDa) was utilized for PEGylation of our gold nanoparticles. We update the technology to be able to enhance bioavailability at the designated target location by carrier surface functionalization.

We acknowledge our study has certain limitations. When comparing the effects between the different cell models, we could not identify a directly proportional relationship between the augmented therapy effects of CD133–functionalized drugs compared to native AuNP-CB-839 in association with the percentage of CD133+cells. We believe this is due to the intrinsic biological heterogeneity of the different tumors the cell models are derived from, which translates into differences in therapy resistance and resistance to GLS1 inhibition. This is supported by our previous studies revealing that GBM1 model is particularly sensitive to CB-839 treatment [12,13]. In this work, we also found that the augmented effect of CD133-functionalized AuPEG-CB-839 is most prominent in this sensitive cell model; let us speculate that enhanced delivery becomes most obvious in this “low resistant cells”. As we show that high GLS1 baseline expression is an indicator of sensitivity to pharmacological approaches, it would be interesting to assess the therapeutic target activity after CD133-AuPEG_CB-839 inhibition. However, it is promising to see that our drug modification augments the therapeutic potential of CB-839 across different functional models, representing different transcriptional subtypes of glioma [49], as GBM1 and U87 are classical subtypes and NC644 is a proneural subtype [50]. Interestingly, the very recent work of Wicker et al. revealed the radiosensitizing potential of CB-839 in the context of head and neck cancer [51]. Given the current standard of care for glioma involves fractionated radiotherapy [52], and given the known fact of high radiation resistance of GSCs and GBM cells at the invasive front of the tumor [53,54], we speculate that the developed nano-carrier might be particularly relevant as an adjuvant treatment to augment the therapeutic potential of current first-line clinical therapy for GBM.

We are aware that our data—at this point—is of correlative nature at most: CD133-positive cell models are more prominently impaired in their growth and viability when treated with the anti-CD133 functionalized drug as compared to when treated with the individual components of the nanomedicine (naïve CB-839 and gold nanoparticles, respectively) only. Further studies, with purified cell population fractions of CD133-positive vs. CD133-negative cells from an isogenic background, either by sorting or genetic enforcement, and comparing the therapeutic effects of the developed nanodrug are needed to prove its targeting efficacy and evaluate the off-target potential in those cells. However, we believe our work provides the essential foundation to do so in follow-up experiments, which shall also include in vivo trials in xenograft models to verify augmented BBB crossing as a result of our drug modification alongside preferential eradication of the CSC population. Given the wide prevalence of CSCs and glutaminolysis-driven malformation in general oncology, we hypothesize that the results may be relevant for stakeholders beyond the neuro-oncology sector.

## 4. Materials and Methods

### 4.1. Study Design

**Figure 9:**
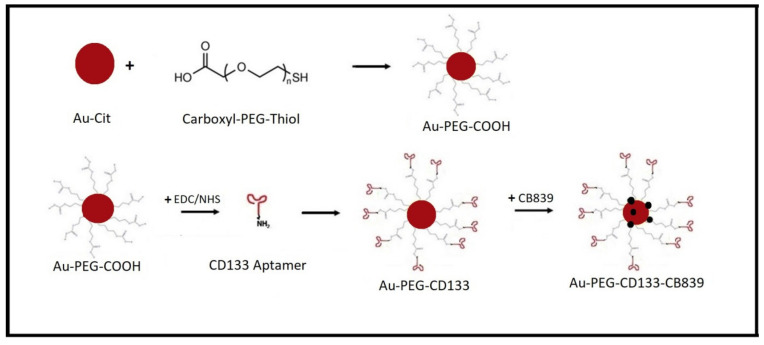
Schematic overview of nanoparticle preparation.

To improve the efficacy of CB-839 as an inhibitor, a PEGylated gold nanoparticle was conjugated with CD133 aptamer-based on carbodiimide chemistry between COOH of carboxy-PEG-thiol and the NH_2_ group of the aminated aptamer. As shown in Figure 1, CB-839 was then loaded into this nanoparticle, and the size of the particle was measured by TEM. In the next step, GSCs and GBM cells were cultured and treated with Nano-medicine. To determine the anti-tumor efficacy of Au-PEG-CD133-CB-839, cell viability was assessed in vitro (Figure 9).

### 4.2. Carboxy-PEG-Thiol Coated Gold Nanoparticle (Au-PEG-COOH) Synthesis

Regarding the protocol routine at the lab for developing gold nanoparticles, which was utilized in the previous study [13] developing and pegylation of gold nanoparticles has been done. Briefly, 19.6 mg of KAuCl_4_ was dissolved in 200 mL of H_2_O and heated to 150 °C on the stirrer at 300 rpm. After boiling the solution, 91.7 mg of sodium citrate was added to change the color to bright red after 15 min. Then carboxy-PEG-thiol (Sigma, Darmstadt, Germany) MW = 5000 in excess (100 µmol) was added and stirred for 24 h. Then we used centrifugation (20,000 rcf, 1 h, 4 °C) for 20 min and suspended in the amount of H_2_O.

### 4.3. Conjugation of CD133 Aptamer to Au-PEG-COOH (Au-PEG-CD133)

In this stage, randomized conjugation of aminated CD133 15mer aptamers (5′-NH_2_-CCCUCCUACAUAGGG-3′) (Eurofins, Hamburg, Germany) occurred with expected free COOH of carboxy-PEG-thiol, which was bound with Au-Cit. In this regard, NaHCO3 solution with pH 7.4 mixed with DMSO in a ratio of 50:50 as a solution of the reaction in Au-PEG-COOH, then EDC (Sigma, Darmstadt, Germany) (10 mg/mL) and sulfoNHS (Sigma, Darmstadt, Germany) (16 mg/mL) were added, respectively, to the stirring solution for 30 min to activate COOH functional groups. Then, activated Au-PEG was mixed with 20 µL of aptamer solved previously in NaHCO_3_ pH: 8.5 and kept for 2 h along with then 10 µL of TEA in the shaker incubator at 37 °C, 350 rpm for 2 h. The Au-PEG-CD133 was centrifuged (22,000 rcf, 1 h, 20 °C) and suspended in DMSO. The result has shown an expected shift based on the covalent reaction between COOH of PEG and NH_2_ of the aptamer.

### 4.4. Loading CB-839 to Au-PEG-CD133

A total of 1 g/L stock of CB-839 was prepared in DMSO based on the amount of CB-839, which was used in the previous study [13], and solving the preparation in DMSO regarding the manufacturer’s suggestion. In addition, the appropriate and suitable DMSO for cell culture was obtained from the biological lab. The solution was heated to 40 °C for 1 h and then regarding the amount of our sample, a specific substance from the stock was mixed with Au-PEG-C133 in order to reach the concentration of 0.05 g/L. Then, the solution was stirred for 4 h for loading the substance. After centrifugation for 1 h at 20,000 rcf and 22 °C, the supernatant was collected to quantify the unloaded amount of CB-839 remaining in the solution via UV-Vis compared to the amount of CB-839 in the stock of 0.05 g/L.

### 4.5. Glioblastoma Stem Cell and Cell Line Culture

GBM1 cells were provided by A. Vescovi (Milan, Italy) and NCH644 were provided by C. Herold-Mende (Heidelberg, Germany) and cultured in DMEM High Glucose (4.5 g/L) without pyruvate, 1 × B27 supplement, 30% Ham’s F12 Nutrient Mix, 20 ng/mL human epidermal growth factor, 20 ng/mL human basic fibroblast growth factor, 5 µg/mL heparin and antibiotic-antimycotic solution. LN229 and U87 cells (CVCL_0022, U-87MG ATCC), kindly provided by A. Weyerbrock (University Freiburg, Freiburg, Germany), were cultured in DMEM (4.5 g/L glucose level) supplemented with 10% FBS (Thermo Scientific, Dreieich, Germany). All cells were cultured in the presence of 1% Penicillin/Streptomycin at 37 °C with 5% CO_2_. The absence of mycoplasma contamination was tested for all cells and their genetic identity was validated using a short tandem repeat analysis. Cells were incubated at 37 °C and 5% CO_2_ for reaching cell cultures to more than 70% confluency and were prepared for different studies. Ethical approval to conduct the in vitro studies was granted by the ethics committee of the Medical Faculty of the Heinrich-Heine University (study ID 5841R).

### 4.6. Fluorescence-Activated Cell Sorting (FACS) Analysis

The direct flow cytometry protocol from Abcam was performed according to the manufacturer’s protocol. In brief, 5–10 × 10^5^ cells were collected and washed twice with cold 10% FBS in PBS, followed by 30 min incubation with the fluorescent-labeled CD133/1 Antibody (130-113-108, Miltenyibiotec, Bergisch Gladbach, Germany) on ice. Cells were washed three times with cold PBS and re-suspended in 500 µL 10% FBS in PBS. Isotype IgG was used as a control. Samples were analyzed within one hour on FACSCanto^®^ (BD, Heidelberg, Germany) or the Guava Muse Cell Analyzer (Luminex, Austin, TX, USA). FSC/SSC exclusion of debris and duplets was done according to general FACS guidelines to focus our analysis on the vital cell populations. Bivariate histograms and statistical plots were generated by the BD FACSDiva^®^ Software at the core facility setup or using web-based Floreada.io software (USA).

### 4.7. MTT Assay and Determination of the Half-Maximal Inhibitory Concentration (IC50)

Glioblastoma models were seeded out in a 96-well culture plate overnight at 37 °C (10,000 cells/well) before starting the treatment experiment the next day, MTT was directly incubated in the 96-well in a final concentration of 0.5 mg/mL and incubated at 37 °C for 90 min. Precipitated formazan crystals were then solubilized with acidified isopropanol (4 mM HCl) containing 1% Triton X-100 and the absorbance was measured at 570 nm (reference 650 nm) using a photometer (Bio-Rad). Statistical analysis and visualization were performed using Graphpad Prism. The dilution series were performed to reach the following final concentration for all treatments conditions (200, 100, 50, 20, and 1 nM). The cells were incubated with the NPs or control compounds followed by measuring cell viability using an MTT assay. We chose this relatively short incubation time due to the immense growth speed of the used cells (doubling time is less than 24 h in our hands). MTT assay was utilized for the evaluation of the cell viability of the nanomedicines (Au-PEG-CD133, Au-PEG-CD133-CB-839).

### 4.8. Statistical Analyses

Analyses were accomplished by using Prism version 6 and all data are shown as mean ± SD. Statistical differences between the groups were evaluated by one-way ANOVA.

## Figures and Tables

**Table 1:** The percentage of CD133 expression in different tumor cell lines.

	CD133 Expression [%]
U87	21.2
LN229	33.8
GBM1	33.1
NCH664	89.2

## Data Availability

All data will be made available upon reasonable request.

## Supplementary Materials

The following are available online at https://www.mdpi.com/article/10.3390/ijms23105479/s1.

## Abbreviations

AuNPs	Gold nanoparticles
BBB	Blood-brain barrier
DLS	Dynamic light scattering
DMSO	Dimethyl sulfoxide
EDC	1-Ethyl-3-(3-dimethylaminopropyl) carbodiimide
DLE	Drug loading efficiency
GBM	Glioblastoma
GLS1	Glutaminase 1
GSCs	Glioblastoma stem cells
MTT	Thiazolyl blue tetrazolium bromide
PEG	Polyethylene glycol
PVA	Polyvinyl alcohol
SulfoNHS	N-hydroxysulfosuccinimide
TEA	Triethylamine
TEM	Transmission electron microscopy
