# Supplementary material for: CD133-Functionalized Gold Nanoparticles as a Carrier Platform for Telaglenastat (CB-839) against Tumor Stem Cells
Source: Int J Mol Sci. 2022 May 13;23(10):5479. doi: 10.3390/ijms23105479 (PMC9141725; doi:10.3390/ijms23105479)
Supplement: Supplementary file 1 [file ijms-23-05479-s001.zip › ijms-1695043-supplementary.pdf]

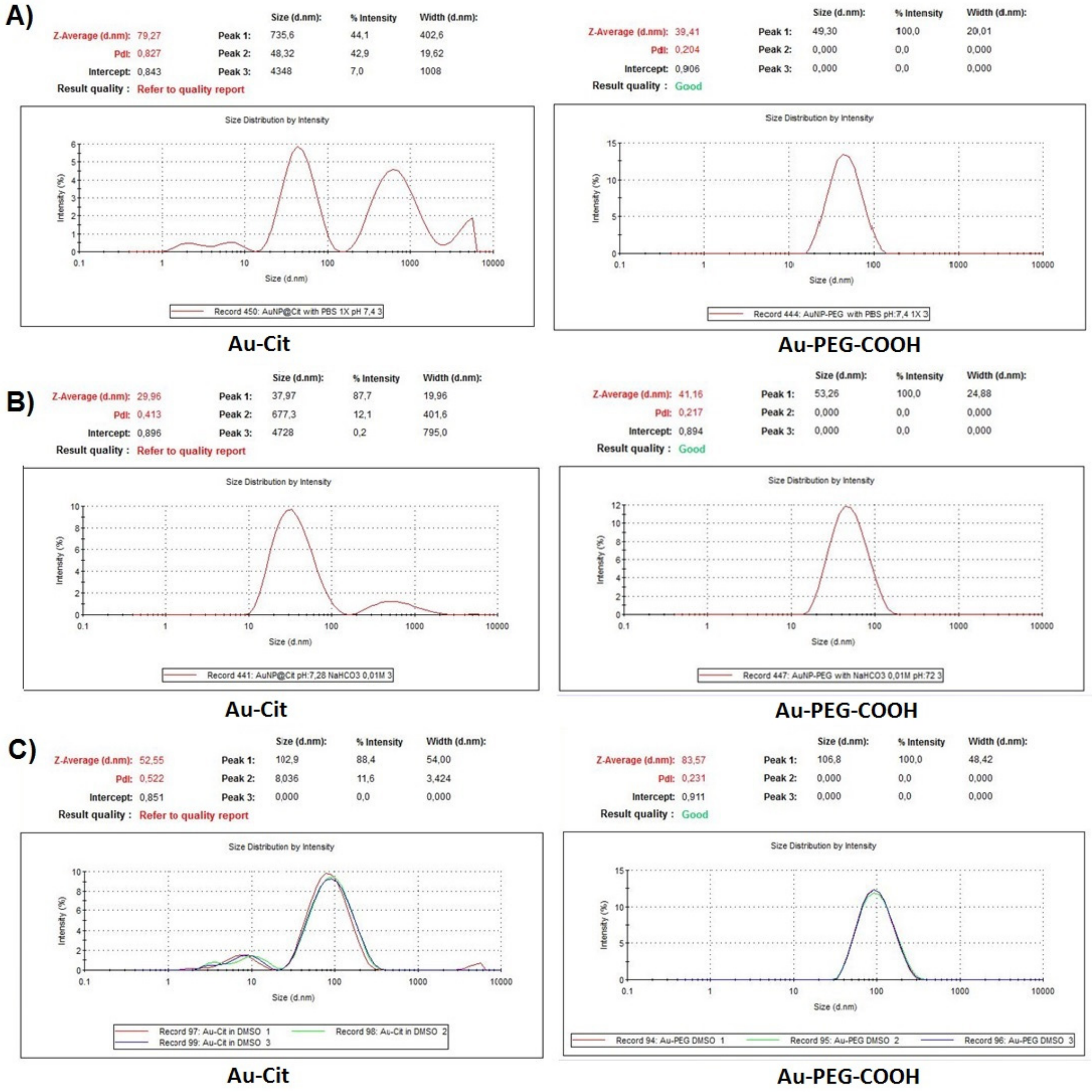


**Figure S1.** Comparison DLS study of the stability of Au-Cit and Au-PEG-COOH in (**A**) PBS 1x pH 7.4, (**B**) NaHCO3 pH 7.2, and (**C**) DMSO. In A, it is evident that the PEGylated gold nanoparticles can be stable in PBS at pH 7.4 as an isotonic solution that is used in biological research whereas Au-Cit shows significant aggregation. In B, both Au-Cit and Au-PEG-COOH are unaffected by NaHCO3 at pH 7.2. On the other hand, in C, both Au-Cit and Au-PEG-COOH indicate some aggregation to particles with four times or two times the average diameter in the aqueous solution (Figure 1 in the main text). This indicates some higher stability of the PEGylated gold nanoparticles compared to Au-Cit in DMSO solution.
